# Draft Genome Sequence of *Lactobacillus crispatus* 2029

**DOI:** 10.1128/genomeA.01221-13

**Published:** 2014-02-20

**Authors:** Andrey V. Karlyshev, Vyacheslav G. Melnikov, Valentin C. Khlebnikov, Vyacheslav M. Abramov

**Affiliations:** a School of Life Sciences, Faculty of Science, Engineering and Computing, Kingston University, Kingston upon Thames, United Kingdom; b International Science and Technology Center, Moscow, Russia; c Institute of Immunological Engineering, Lyubuchany, Moscow Region, Russia

## Abstract

This report describes a draft genome sequence of *Lactobacillus crispatus* 2029. The reads generated by the Ion Torrent PGM were assembled into contigs with a total size of 2.2 Mb. The data were annotated using the NCBI GenBank and RAST servers. A comparison with the reference strain revealed specific features of the genome.

## GENOME ANNOUNCEMENT

*Lactobacillus crispatus* strain 2029 was isolated in 2011 at the Institute of Engineering Immunology, Lyubuchany, Russia, upon investigation of a bacterial species spectrum of vaginal lactobacilli. Among 726 strains of *L. crispatus* tested, only 19 strains were able to produce hydrogen peroxide at a level of 100 to 120 mg/liter. Only three of these strains were found to be resistant to intestinal stress conditions. Of these, strain 2029 was the only one to satisfy the technological requirements. This strain was found to be able to adhere to vaginal and intestinal epithelial cells. Strain *L. crispatus* 2029 is deposited at the All-Russian Collection of Microorganisms at the G. K. Skryabin Institute of Biochemistry and Physiology of Microorganisms (Russian Academy of Sciences, Pushchino, Russia) under registration no. VКМ В-2727D.

Here, we present a draft genome sequence of strain *L. crispatus* 2029. The only complete *L. crispatus* genome sequence available at the time of preparation of this paper was that of strain ST1 (1). The sequencing reads, generated by IonTorrent PGM using Chip 314, were assembled by Ion Torrent assembler into 295 contigs (2.0 to 40.8 kb). The total size of the assembly (2,193,123 bases, 36.08-fold genome coverage) and G+C content (36.9%) are in full agreement with the published data for other strains of *L. crispatus* (2.04 to 2.46 Mb and 36.7 to 37.1%).

The genome sequence was annotated using NCBI GenBank and RAST (2) genome annotation servers. Among the genes found were those required for bacteriocin biosynthesis and transport. Several S-layer protein-coding genes were detected, along with a gene encoding a fibronectin-binding protein (FBP). In contrast to *L. crispatus* ST1, a gene encoding a mucus binding protein was absent. Homologues to only four out of S-layer proteins, encoded by strain ST1, were present. A gene encoding a homologue of streptolysin S biosynthesis protein SagB was present in the test strain but not in the reference strain ST1.

One gene product was annotated by both programs as “lactocepin S-layer protein,” with a predicted size of 158 amino acids. A similar protein is also encoded by *L. crispatus* ST1 (158 amino acids [aa] long, 99% identity). In general, lactocepins are cell surface-located or -secreted peptidases responsible for anti-inflammatory effects via selective degradation of proinflammatory chemokines (3). However, these proteins are significantly larger in size, e.g., the originally described lactocepin produced by *L. lactis* is 1,715 amino acids long (4). It remains to be elucidated whether the *L. crispatus* 2029 protein annotated as lactocepin has a similar function and activity.

The sequencing data were also analyzed by mapping reads onto the reference genome of strain ST1 using the CLC Genomics Workbench software. NCBI BLASTx analysis of assembled unmapped reads identified the genes present in the test strain genome but not in the reference genome. Among these genes were those encoding a pullulanase, phage-related proteins (integrases and resolvases), transposases, and a CRISPR-associated protein.

The draft genome sequence of *L. crispatus* described in this report will assist in the understanding of specific features of this strain as a promising probiotic.

### Nucleotide sequence accession numbers.

This whole-genome shotgun project has been deposited at DDBJ/EMBL/GenBank under the accession no. AVFH00000000. The version described in this paper is version AVFH02000000.
